# SpineML and Brian 2.0 interfaces for using GPU enhanced Neuronal Networks (GeNN)

**DOI:** 10.1186/1471-2202-15-S1-P148

**Published:** 2014-07-21

**Authors:** Thomas Nowotny, Alexander J Cope, Esin Yavuz, Marcel Stimberg, Dan FM Goodman, James Marshall, Kevin Gurney

**Affiliations:** 1 CCNR, School of Engineering and Informatics, University of Sussex, Falmer, Brighton BN1 9QJ, UK; 2 Department of Computer Science, University of Sheffield, Sheffield S1 4DP, UK; 3 Institut d’Etudes de la Cognition, Ecole Normale Supérieure, Paris, France; 4 Harvard Medical School, Harvard University, Boston, MA 02115, USA; 5 Department of Psychology, University of Sheffield, Sheffield S10 2TP, UK

## Background

The GPU enhanced Neuronal Networks (GeNN) framework [1,2] was introduced in 2011 to facilitate the efficient use of graphical processing units (GPUs) as accelerators for neuronal network simulations, in particular as part of computational neuroscience investigations. GeNN is based substantially on code generation for the NVIDIA CUDA application programming interface. Code generation provides decisive advantages over stand-alone simulators in that (i) code can be optimized both for every individual model and for the specific GPU hardware detected at compile time, and (ii) code generation allows to provide a practically limitless number of pre-defined models while the generated simulation code remains as small and efficient as possible.

While GeNN is an important step towards facilitating the use of GPU acceleration for computational neuroscience applications it has been designed with expert users in mind. Particular emphasis has been put on flexibility and extendibility and, in case of conflict, these were prioritized over the easy of use.

In the work presented here we are aiming to make GeNN and the corresponding GPU acceleration now also available to non-expert users by providing two new interfaces from SpineCreator [3]/SpineML [4] and the Brian 2 simulator [5,6] to GeNN.

## Results

We have created prototype interfaces from SpineCreator using the SpineML markup language and from Brian 2 by modifying the code generation facilities within Brian 2 to generate valid GeNN input files. In both cases, a middleware was created that takes the model descriptions that were either available in SpineML (as generated by SpineCreator) or the internal representation in Brian 2 and translate them into the three main code parts necessary to run a GeNN simulation: (i) Neuron, synapse and network definitions including variables, parameters, code to integrate dynamical equations and properties of the connectivity, (ii) Code that runs the GeNN generated code to execute the simulation and (iii) code that regulates the transfer of information from SpineCreator/Brian 2 to GeNN and vice versa (e.g. for providing connectivity matrices and returning simulation results).

## Conclusions

We believe that when the new SpineCreator and Brian 2 interfaces will have been fully developed, tested and released we can make a decisive difference in the uptake of GPU acceleration for neuronal network simulations. The completed prototypes show sufficient flexibility and ease of use to be appealing to a wide range of future users.
